# The self-organization model reveals systematic characteristics of aging

**DOI:** 10.1186/s12976-020-00120-z

**Published:** 2020-03-20

**Authors:** Yin Wang, Tao Huang, Yixue Li, Xianzheng Sha

**Affiliations:** 1 Department of Biomedical Engineering, School of Fundamental Sciences, China Medical University, Shenyang, 110012 Liaoning Province China; 2 Tumor Etiology and Screening Department of Cancer Institute and General Surgery, The First Affiliated Hospital of China Medical University, 155# North Nanjing Street, Heping District, Shenyang City, 110001 Liaoning Province China; 3 Shanghai Institute of Nutrition and Health, Shanghai Institutes for Biological Sciences, Chinese Academy of Sciences, Shanghai, 200031 People’s Republic of China; 4 Bio-Med Big Data Center, Key Laboratory of Computational Biology, CAS-MPG Partner Institute for Computational Biology, Shanghai Institute of Nutrition and Health, Shanghai Institutes for Biological Sciences, University of Chinese Academy of Sciences, Chinese Academy of Sciences, Shanghai, 200031 China; 5 School of Life Sciences and Biotechnology, Shanghai Jiao Tong University, Shanghai, 200240 China; 6 Collaborative Innovation Center of Genetics and Development, Fudan University, Shanghai, 200433 China; 7 Shanghai Center for Bioinformation Technology, Shanghai Academy of Science and Technology, Shanghai, 201203 China

**Keywords:** Aging, Supervised learning, Self-organization, Network analysis

## Abstract

**Background:**

Aging is a fundamental biological process, where key bio-markers interact with each other and synergistically regulate the aging process. Thus aging dysfunction will induce many disorders. Finding aging markers and re-constructing networks based on multi-omics data (i.e. methylation, transcriptional and so on) are informative to study the aging process. However, optimizing the model to predict aging have not been performed systemically, although it is critical to identify potential molecular mechanism of aging related diseases.

**Methods:**

This paper aims to model the aging self-organization system using a series of supervised learning methods, and study complex molecular mechanisms of aging at system level: i.e. optimizing the aging network; summarizing interactions between aging markers; accumulating patterns of aging markers within module; finding order-parameters in the aging self-organization system.

**Results:**

In this work, the normal aging process is modeled based on multi-omics profiles across different tissues. In addition, the computational pipeline aims to model aging self-organizing systems and study the relationship between aging and related diseases (i.e. cancers), thus provide useful indicators of aging related diseases and could help to improve prediction abilities of diagnostics.

**Conclusions:**

The aging process could be studied thoroughly by modelling the self-organization system, where key functions and the crosstalk between aging and cancers were identified.

## Introduction

Aging is a complex process regulated by key bio-markers, reflecting disorders / declined abilities of tissues [1]. Dysfunction of aging has been shown to be related to many diseases, such as diabetes, Parkinson disease [2], Alzheimer’s disease [3] and cancers [4]. As a result, finding aging markers is critical to study aging related diseases and identify healthy genomic diagnostics (i.e. by predicting the chronological age (group) based on molecular profiles). For example, multi-tissue predictors of age have been calculated by DNA methylation [5] or mRNA expression profiles [6]; and there are more age predictors based on single tissue (e.g. brain [7], breast [8], ans so on), which also provide insights on aging related diseases [6] (i.e. Alzheimer’s disease and cancers).

Further, the aging markers interact with each other [9], and synergistically coordinate the aging process, herein generating the self-organization system [10] of aging, where particular bio-markers regulate the aging process in different age groups, respectively. Although tissues become disordered reflected by a functional decline during aging in general (often evaluated by increased entropies [11]), a series of aging markers perform particular / ordered functions in special aging stages / age groups. In addition, these markers / genes interact with each other, coordinating regulation of the aging process; therefore, the interactions / modules between such markers also provide critical patterns of aging. In summary, finding bio-markers to predict the chronological ages, summarizing interactions between aging markers, and optimizing the aging self-organization system based on molecular profiles (i.e. methylation, expression and so on) of normal tissues from healthy persons, could help predict future health risks at system level. However, these works have not been solved entirely for the aging process.

In this work, we modeled the aging self-organization system using a series of computational methods: filtering inter-connection networks between different age groups by the maximum mutual information and minimum redundancy criterion in the information theory; summarizing interactions between bio-markers by the convolution technology; calculating patterns by accumulating weighted genes within the same module; selecting module scales by the hierarchical clustering method and cross validation; identifying order parameters in the aging self-organization system by network sparsification.

The prediction results show high classification accuracy between different age groups; moreover, the enrichment analysis and network analysis also found key functions of the order parameters. Thus critical complex characteristics (i.e. hierarchies, emergencies and bifurcations) were identified in different aging stages. Aging acceleration patterns were also identified across cancers. In short, the aging process can be thorough studied by modelling the self-organization system.

## Results and discussion

### A brief description of the aging self-organization system

In the aging self-organization system, genes interacted with each other, and synergistically coordinated the aging process. Therefore, the aging process should also be evaluated by interactions between aging markers. The aging markers clustered nearby would drive similar function [12], and could be summarized within the same module. Each module takes a particular part during aging, and regulates the aging process altogether. Different levels / hierarchies of aging markers or modules reflected special complexities of the self-organization system, herein reflecting particular patterns in different aging stages. As a result, the aging self-organization system would identifies and emphasizes important characteristics that no single isolated marker or module would be able to achieve. In summary, modules based on aging markers in coordination determined the bifurcations and displayed critical differential patterns between age groups, where key aging markers within modules could be identified as the order parameters in the aging self-organization system.

**Fig. 1:**
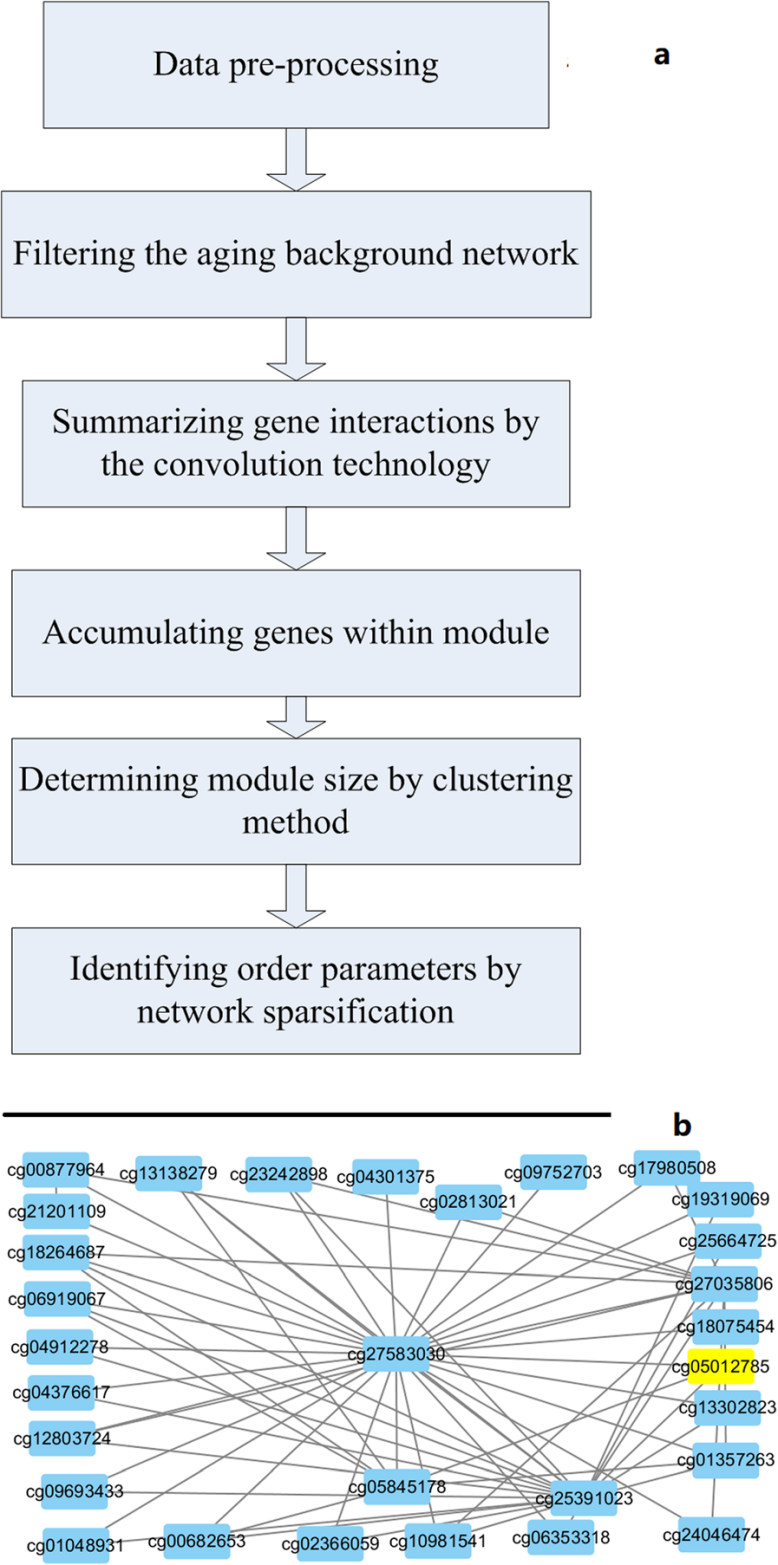
Overview of the aging self-organization system. **a** the computational pipeline of modelling the aging self-organization system; **b** an example of the convolution of interactions between methylation cg27583030 (SLC25A4) and other genes in the model of age group 50–70 vs. 70-survival

Further, the system from pathological samples show deviation from the normal aging self-organization system (from healthy persons): for example, the aging system with disease (i.e. cancers) should display significant acceleration compared tothe normal aging process. Accordingly, the following parts of this paper presents results of modelling the aging self-organization system as well as the computational pipeline was shown in Fig. 1.

### Classification results of the aging self-organization system

**Fig. 2:**
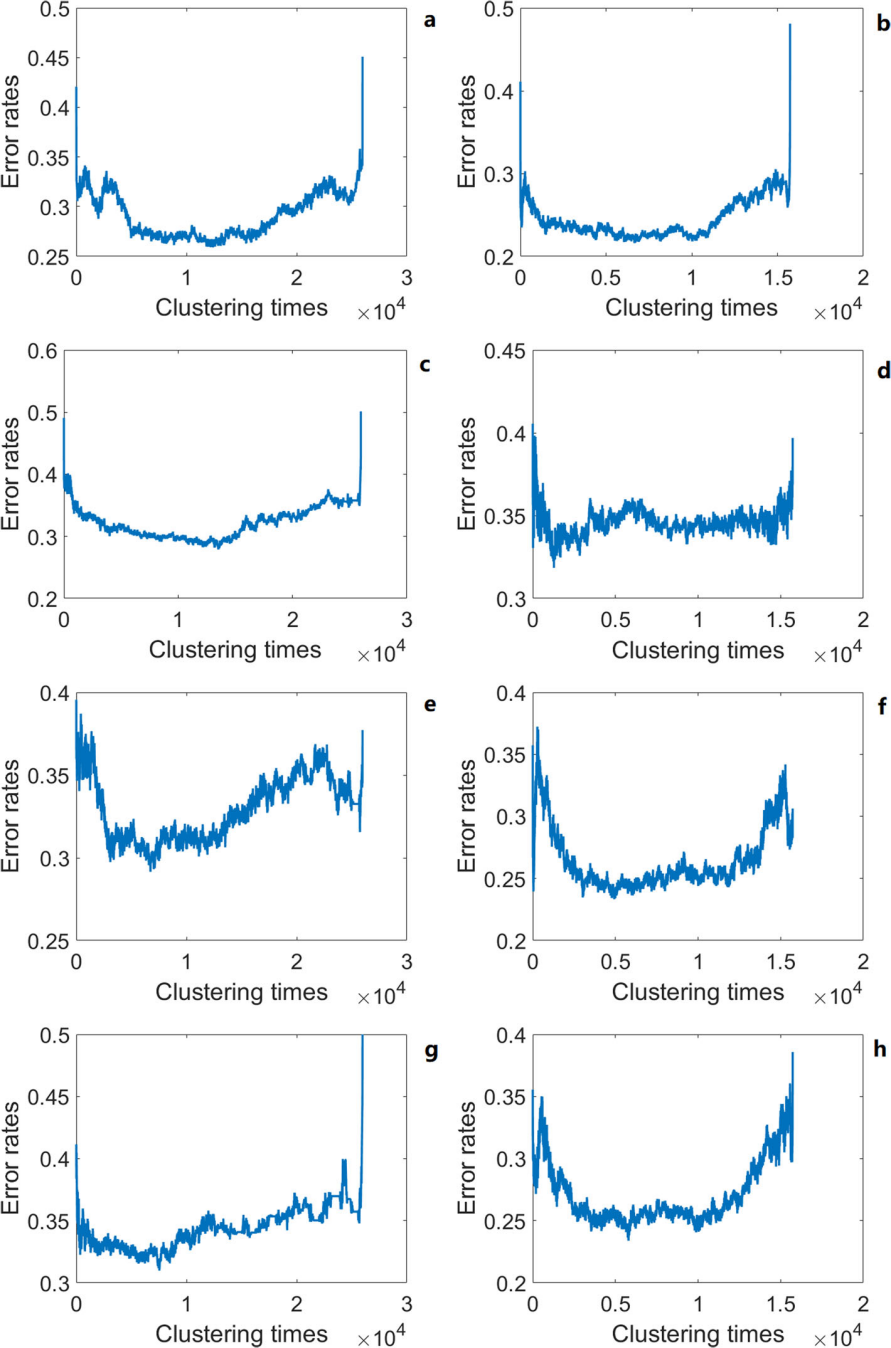
Learning curves of the the aging self-organization system. **a**, **c**, **e**, **g** methylation profiles; **b**, **d**, **f**, **h** expression profiles; **a**, **b** 0–50 vs. 50-survival; **c**, **d** 0–20 vs. 20–50; **e**, **f** 20–50 vs. 50–70; **g**, **h** 50–70 vs. 70-survival

**Fig. 3:**
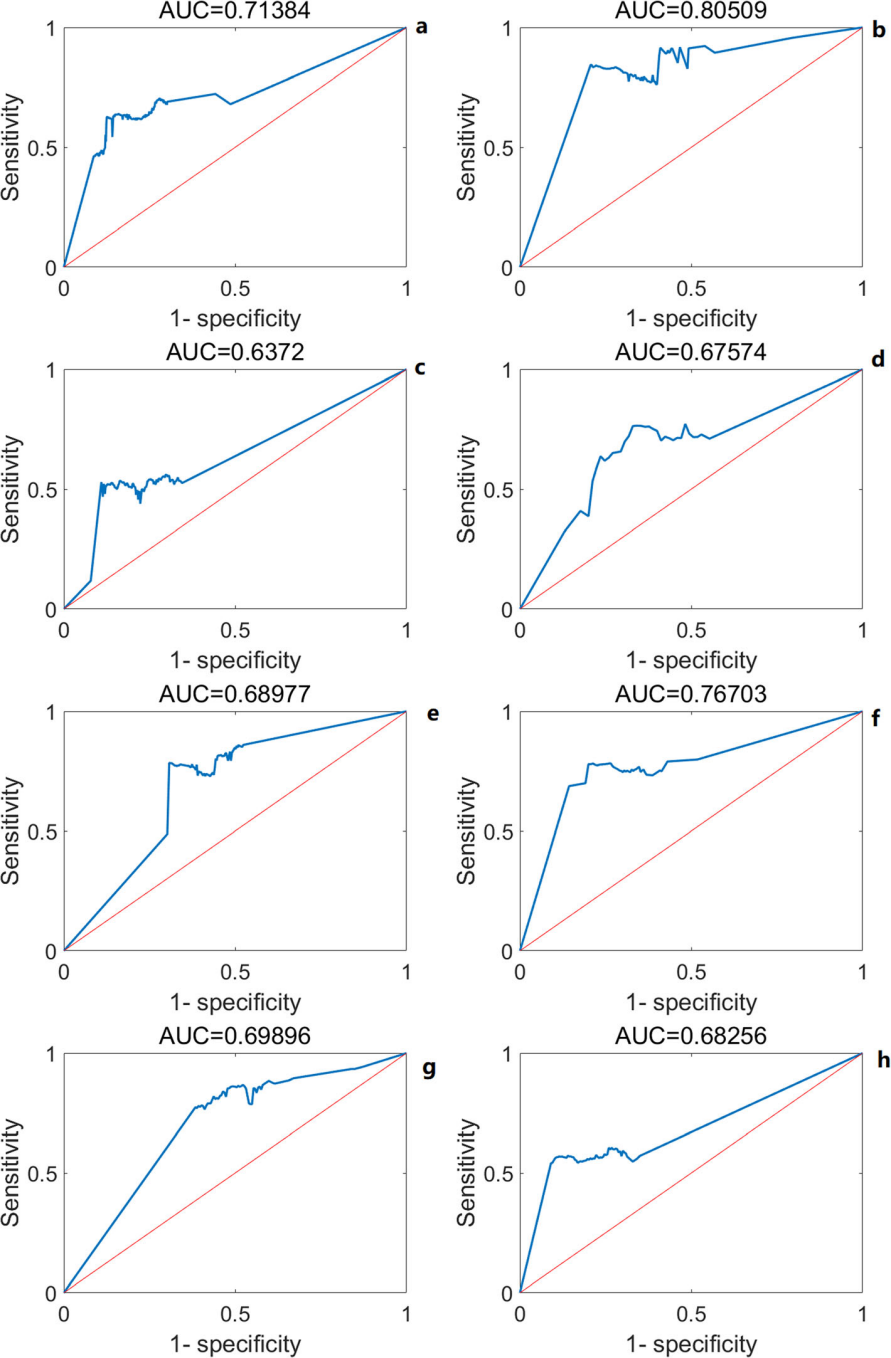
ROC curves of the the aging self-organization system in test data. **a**, **c**, **e**, **g** methylation profiles; **b**, **d**, **f**, **h** expression profiles; **a**, **b** 0–50 vs. 50-survival; **c**, **d** 0–20 vs. 20–50; **e**, **f** 20–50 vs. 50–70; **g**, **h** 50–70 vs. 70-survival;

The expression and methylation profiles were used to test the classification abilities of the aging self-organization system between different age groups, respectively (Figs. 2 and 3). Tables 1, 2 and 3 shows that classification results based on the self-organization system have lower error rates than traditional feature selection methods (i.e. the relieff-mRMR pipeline [13]), using both methylation and expression data between age groups. As a result, the self-organization system reduced the feature dimensions effectively and extracted critical modules by finding order-parameters, and identifying key differences based on aging markers / interactions in the aging process at system level.

**Table 1:** Overview of the aging self-organization system

Model	Profiles	Samples (training data + test data)	Order parameters (original profiles + summarized interactions)	Modules (dimensions)
0–20 vs. 20–50	methylation	1760 + 798	4360 + 6408	2683
20–50 vs. 50–70	methylation	1875 + 845	2137 + 6713	2920
50–70 vs. 70-survival	methylation	1285 + 585	1484 + 5497	1918
0–50 vs. 50-survival	methylation	3045 + 1381	4825 + 4891	2746
0–20 vs. 20–50	expression	655 + 315	246 + 2211	1173
20–50 vs. 50–70	expression	975 + 473	2658 + 4660	2388
50–70 vs. 70-survival	expression	845 + 411	2201 + 6044	2454
0–50 vs. 50-survival	expression	1500 + 726	2832 + 4413	2067

**Table 2:** Classification results (error rates) based on methylation data

Age group	Training data (cross validation)	Test data	Training data (control method)	Test data (control method)
0–20 vs. 20–50	0.279	0.3356	0.44	0.4726
20–50 vs. 50–70	0.2916	0.2719	0.422	0.461
50–70 vs. 70-survival	0.3097	0.3098	0.3262	0.3137
0–50 vs. 50-survival	0.259	0.2549	0.3791	0.4136

**Table 3:** Classification results (error rates) based on expression data

Age group	Training data (cross validation)	Test data	Training data (control method)	Test data (control method)
0–20 vs. 20–50	0.3185	0.3084	0.351	0.3911
20–50 vs. 50–70	0.2333	0.2308	0.2613	0.4325
50–70 vs. 70-survival	0.2337	0.279	0.2726	0.4659
0–50 vs. 50-survival	0.2161	0.2066	0.3492	0.4297

### Biological features of the order parameters

The results of the order-parameters were shown in Table S1-S2. The methylation order parameter with the maximum relieff weight was the convolution interactions of cg27583030 (SLC25A4, weight = 0.1974, shown in Fig. 1b) in the model of age group 50–70 vs. 70-survival. Common SLC25A4 related pathway were apoptosis and survival regulation of apoptosis by mitochondrial protein, reflecting the relationship between aging and cellular apoptosis [14]. The expression order parameters with the largest relieff weight was original profile of NRBF2 (weight = 0.2493) between age group 50–70 vs. 70-sruvival. NRBF2 played a role in cellular survival and neural progenitor cell survival during differentiation [15], and dysfunction of NRBF2 also affected the aging process.

Further, enrichment analyses of the order parameters in each module were performed on Biological Process (BP) terms of Gene Ontology (GO) and KEGG pathways using the hypergeometric test (Tables 4 and 5 and S1-S2). The most significant BP term was the negative regulation of viral process (GO:0048525, fdr = 0.0005) in the 245th module of the model between age group 20–50 vs. 50–70 based on the methylation profiles, reflecting the relationship between the immunity system and aging [16]; and the most significant KEGG pathway was Phenylalanine metabolism (fdr = 0.0009) based on the methylation profiles in the model between age group 0–50 vs. 50-survival, indicating the critical metabolism during aging. In addition, the annotation of order-parameters also reveal key functions across different aging stages, i.e. BP terms were enriched in aging related diseases in the early stage of aging, and enriched in tissue dysfunction in the later stage. It is perhaps indicative of functional decline of the immunity system induced aging / tissue dysfunction.

**Table 4:** Top enriched BP terms within module

Model	Profiles	Pathway	FDR
0–20 vs. 20–50	methylation	negative regulation of cellular senescence (GO:2000773)	0.0016
20–50 vs. 50–70	methylation	negative regulation of viral process (GO:0048525)	0.0005
50–70 vs. 70-survival	methylation	prostage gland morphogenesis (GO:0016578) branch elongation of an epithelium (GO:0060602) axis elongation (GO:0003401)	0.0041
0–50 vs. 50-survival	methylation	histone deubiquitination (GO:0016578)	0.003
0–20 vs. 20–50	expression	response to electrical stimulus (GO:0051602)	0.0324
20–50 vs. 50–70	expression	fatty acid beta-oxidation using acyl-CoA oxidase (GO:0033540) alpha-linolenic acid metabolic process (GO:0036109)	0.0014
50–70 vs. 70-survival	expression	progesterone metabolic process (GO:0042448)	0.0028
0–50 vs. 50-survival	expression	purinergic nucleotide receptor signaling pathway (GO:0035590)	0.0012

**Table 5:** Top enriched KEGG pathways within module

Model	Profiles	Pathway	FDR
0–20 vs. 20–50	methylation	Maturity onset diabetes of the young	0.0019
20–50 vs. 50–70	methylation	Focal adhesion	0.0015
50–70 vs. 70-survival	methylation	Homologous recombination	0.0014
0–50 vs. 50-survival	methylation	Phenylalanine metabolism	0.0009
0–20 vs. 20–50	expression	Lysosome	0.0104
20–50 vs. 50–70	expression	Retinol metabolism	0.0022
50–70 vs. 70-survival	expression	Prion diseases	0.0027
0–50 vs. 50-survival	expression	Long-term potentiation	0.0035

**Fig. 4:**
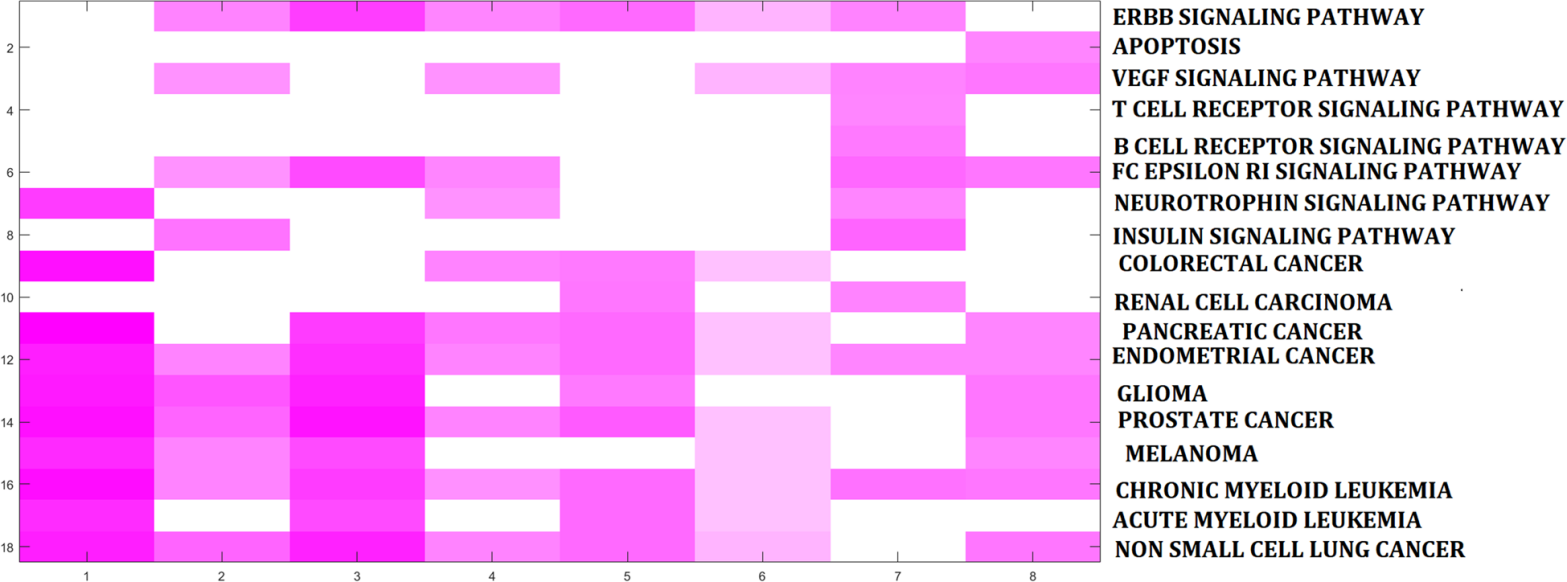
Biological features of the the aging self-organization system. The top 10 enriched KEGG pathways across different models of aging self-organization systems

Strikingly, enriched functions across modules indicated the common themes of aging (Tables 6 and 7). For example, BP terms of organ morphogenesis were enriched during both young (0–20 vs. 20–50) and old (50–70 vs. 70- survival) age groups, reflecting the basal role of tissues affected by the aging process. Moreover, cancer and related signaling KEGG pathways were enriched across different aging stages based on both methylation and expression profiles (Fig. 4). These results reflected the cross-talk between aging and cancer, where dysfunction of aging might indicate diseases / cancerization of tissues.

**Table 6:** Top enriched BP terms across modules

Model	Profiles	Pathway	FDR range	Enriched modules	Score
0–20 vs. 20–50	methylation	outflow tract morphogenesis (GO:0035902) cardiac septum morphogenesis (GO:0060411)	0.0409~0.0614	2	1.8977
20–50 vs. 50–70	methylation	negative regulation of viral process (GO:0048525)	0.0061~0.1663	2	1.8276
50–70 vs. 70-survival	methylation	developmental growth involved in morphogenesis (GO:0060560)	0.0266~0.0845	3	2.8406
0–50 vs. 50-survival	methylation	response to immobilization stress (GO:0035902)	0.0031~0.0937	2	1.9031
0–20 vs. 20–50	expression	obsolete regulation of cyclic nucleotide metabolic process (GO:0030799)	0.0791~0.0867	2	1.8342
20–50 vs. 50–70	expression	regulation of response to external stimulus (GO:0032101)	0.0752~0.1723	3	2.5805
50–70 vs. 70-survival	expression	cardiac septum development (GO:0003279)	0.0358~0.0915	2	1.8725
0–50 vs. 50-survival	expression	neural tube formation (GO:0051602)	0.0333~0.0775	2	1.8892

**Table 7:** Top enriched KEGG pathways across modules

Model	Profiles	Pathway	FDR range	Enriched modules	Score
0–20 vs. 20–50	methylation	Glioma	0.0193~0.184	11	9.8185
20–50 vs. 50–70	methylation	Prostage cancer	0.0922~0.185	16	13.6894
50–70 vs. 70-survival	methylation	Pancreatic cancer	0.0714~0.1931	9	7.9217
0–50 vs. 50-survival	methylation	Pancreatic cancer	0.041~0.1988	17	14.7595
0–20 vs. 20–50	expression	ERBB signaling pathway VEGF signaling pathway Non-small cell lung cancer	0.0902~0.1844	5	4.3889
20–50 vs. 50–70	expression	Insulin signaling pathway	0.0213~0.1858	10	8.8731
50–70 vs. 70-survival	expression	VEGF signaling pathway Fc epsilon RI signaling pathway	0.0134~0.194	9	7.8764
0–50 vs. 50-survival	expression	Prostage cancer	0.0692~0.1887	11	9.4625

### Complexity characteristics of the aging self-organization system

In the self-organization system, molecules interacted with each other, and synergistically regulate particular process (i.e. aging). For example, summarized interactions of methylation profiles of cg27583030 (SLC25A4) was with the maximum relieff value (Fig. 1b). Moreover, a series of order parameters were with summarized interactions other than the ordinary profiles, indicating functions of cross-talks between key markers during aging (Table 1).

In addition, any single isolated marker / module could not reflect the whole bifurcation between age groups effectively, but combinations of modules could. It should be that cross-talks across modules generated the aging self-organization system with key differential patterns / bifurcations between age groups. Thus the hierarchies across modules promoted emergencies of the aging self-organization system, which were not displayed by any single module or order parameter. Therefore, the interaction across modules indicated the hierarchies / emergence of the aging self-organization system.

The corresponding networks across modules (using the maximum mutual information minimum redundancy criterion) were investigated to find core modules in the hierarchies of the self-organization system (Figure S1). Based on the methylation profiles, the 492th module were with the maximum interaction score (mean value = 0.0189), connecting other 2682 modules (in the model of age group: 0–20 vs. 20–50). This module acted as a “hub” and connected 45 key BP functions (i.e. negative regulation of cellular senescence, regulation of attachment of spindle microtubules to kinetochore and negative regulation of potassium ion transmembrane transporter activity) and 60 KEGG pathways (i.e. Maturity onset diabetes of the young and Pentose and glucuronate interconversions), reflecting the crosstalk between immunity and key metabolic pathway during aging. Based on the expression profiles, the 1799th module were with the maximum interaction score (mean value = 0.0762), connecting other 2387 modules (in the model of age group: 20–50 vs. 50–70). The module connected 46 key BP functions (i.e. fatty acid beta oxidation using acyl coa oxidase, cell aggregation and cytokine production) and 51 KEGG pathways (i.e. Retinol metabolism, alpha linolenic acid metabolism and Pyruvate metabolism), revealed basal metabolism pathways during aging. In short, the corresponding networks reflected correlations of important parts / functions in the aging self-organization system, such as the immunity system, cancer related pathways, and so on. It might be the cross-talk between the immunity system and cancers that cause of emergence of critical themes in the aging process.

**Fig. 5:**
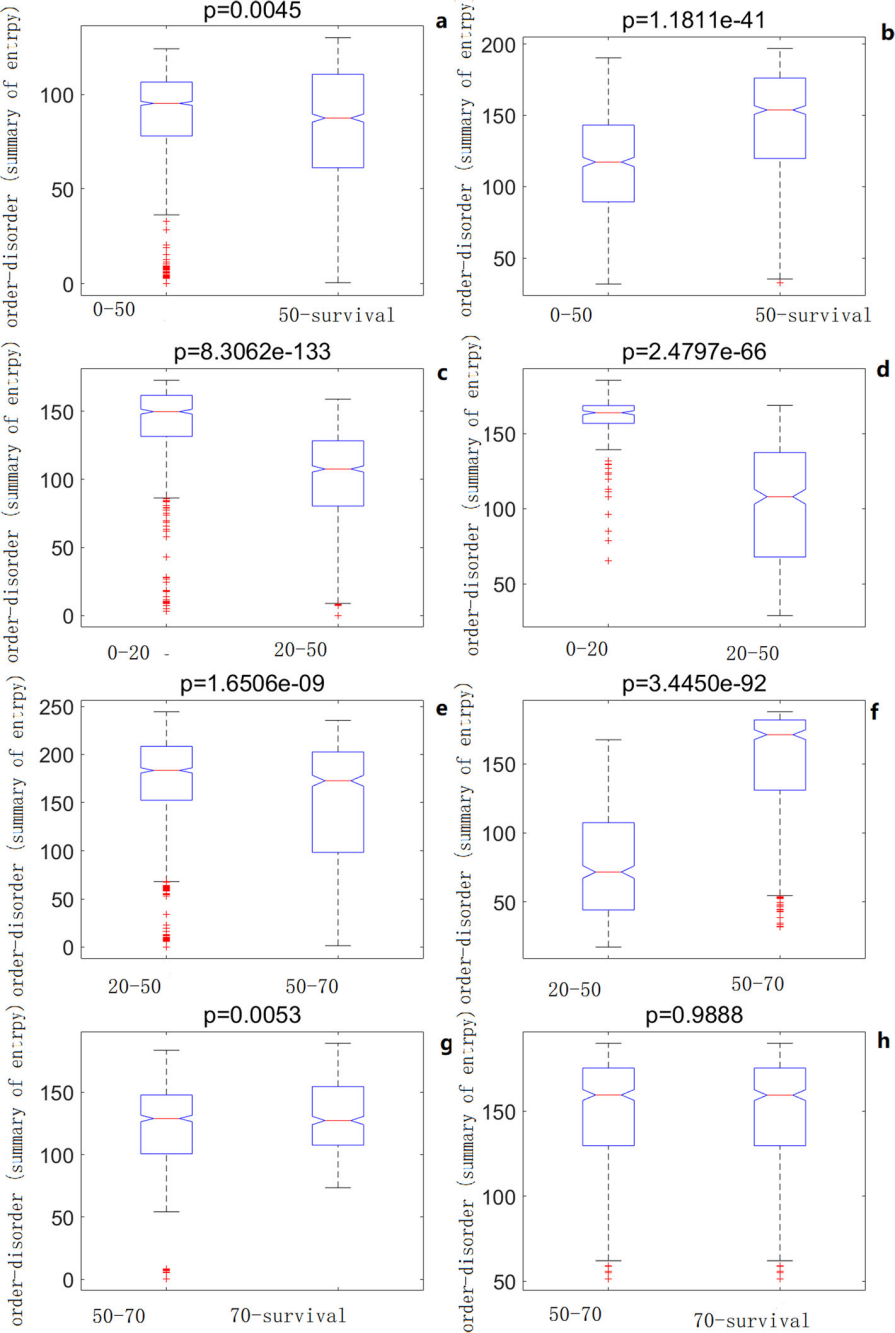
Order-disorder patterns in different aging stages. **a**, **c**, **e**, **g** methylation profiles; **b**, **d**, **f**, **h** expression profiles

Therefore, the bifurcations (differential patterns) between age groups were investigated, where order parameters with convoluted interactions were summarized by each relieff weight, indicating order-disorder patterns of interactions between aging order-parameters from low (near the ordered / similar pattern with low entropic values of the aging system) to high (near disordered / different patterns with high entropic values) values, or vise verse. As a result, significant differential patterns were found between age groups using both methylation and expression profiles (Fig. 5). In the early aging stage (0–20 vs. 20–50), the aging self-organization systems were with significantly ordered patterns based on both methylation and expression profiles. As aging was regulated by special markers / order-parameters, the self-organization system showed ordered patterns in the early aging stage. However, the self-organization systems were with disordered patterns in the middle (20–50 vs. 50–70) or later (50–70 vs. 70-survival) stage based on expression or methylation profiles, respectively. These results indicated tissues were with declined function / disordered patterns during aging. In short, the aging process was driven by both special markers / order parameters (with ordered patterns) and tissue declined function (with disordered patterns), perhaps determined by the former in the early aging stage, and by the latter in the later aging stage.

### Aging acceleration of cancers

**Fig. 6:**
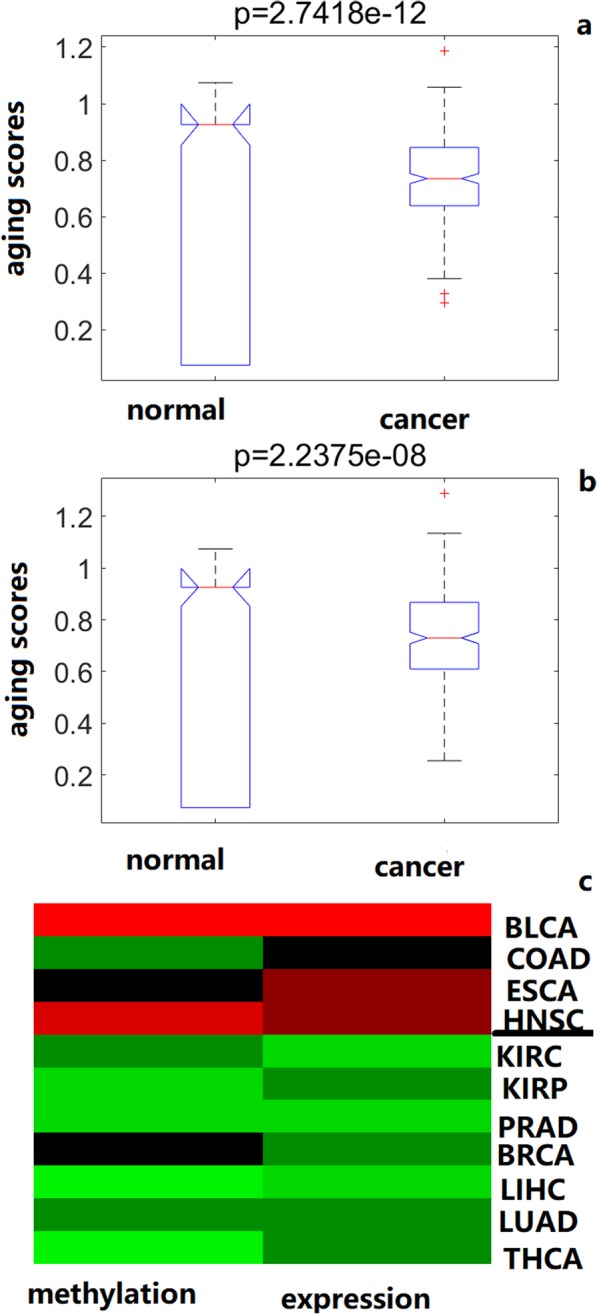
Aging acceleration in cancers. **a** methylation profiles; **b** expression profiles; **c** The heatmap of aging acceleration patterns across cancers

To study the crosstalk between aging and cancer, the aging associated accelerations were investigated in cancer samples (from the TCGA platform). The order parameters were extracted using the cancer profiles based on each self-organization model, and the module patterns in each model were summarized. Then the aging score were calculated by the module patterns (adjacent normal samples were as the training data, cancer samples were as the test data, and the 0–1 SVM regression was used as the predictor). Strikingly, the results showed that the scores in cancer samples were significantly higher compared to adjacent normal samples, based on both methylation and expression profiles (Fig. 6). These results were consistent with previous results, which might reflect the protection of the cancer tissues [5, 17].

The correlation between somatic mutation and aging acceleration was also investigated, but none of SNPs with significant (*p*-value< 0.05 and fdr < 0.2, using the non-parameter Kruskal-Wallis Test [18]) aging acceleration were identified. The results were perhaps because of inadequate samples of paired profiles, aging acceleration tissues in cancer with fewer somatic mutations, or the complexity of aging [5]. Moreover, it has been found that there was negative correlation between age acceleration and number of somatic mutations [5]. Therefore, our work also found the negative correlations in most types of cancers (Figure S2 and S3). However, only a few cancers were with significant correlation (e.g. THCA, shown in Figure S2l and S3l).

Further, the 11 types of cancer profiles were clustered based on the mean value of aging acceleration patterns using mean acceleration ratios based on simplified methylation and expression models. As a result (Fig. 6c and S4), 7 cancers were identified as one aging acceleration pattern, including BLCA, COAD, ESCA, HNSC, KIRC, KIRP and PRAD; and other 4 cancers were identified as another aging acceleration pattern (BRCA, LIHC, LUAD and THCA). In addition, 49 significant modules were identified based on both differential methylation and expression profiles, where the top differential module (the 926th expression module) connected 50 key BP terms (i.e. cellular localization, skeletal muscle adaptation and GABAergic neuron differentiation) and 51 KEGG pathways (Long-term potentiation and Retinol metabolism), indicating key functions of the aging process of the neuron system. The aging acceleration patterns also revealed basal characteristics across cancers.

## Discussion

The aging process is regulated by a series of key markers. The aging markers interact with each other, and performed their functions in the specific aging networks. As a result, identifying modules clustered by these markers during aging was more informative to research the aging process compared to finding isolated markers.

In other words, differential interactions (evaluated by the mutual information) in proper networks were also powerful to study key gene regulations in biological processes (i.e. aging and related diseases) [19]. Both the network markers and gene markers were integrated based on the background networks to identify critical markers in the aging process thereby. Further, these aging markers were extracted as crucial features of aging, where the separation margin of SVM was optimized / converged to discriminate different age groups. As a result, the SVM hyper plane based on extracted network markers (both methylation and expression profiles) had enough efficiency to classify age groups and explorer critical mechanisms of the aging process, compared with other classfiers (Table S3 and S4).

In this paper, we presented a computational pipeline to model the aging self-organization system and select the order parameters using a series of supervised learning technologies. The discrimination results showed that our prediction ability had more accuracy than traditional gene selection / classification methods.

Tissues suffer declined functions during aging. However, the aging markers interact with each other, and synergistically regulate the aging process. Therefore, the aging process is affected by both ordered and disordered factors. In this work, the complexity characteristics across modules were also found to show critical patterns in different age groups.

In the immunity theories of aging [20], tissues exhibit progressively functional declines during aging. The functional analysis found that the order parameters were enriched in particular BP terms / KEGG pathways in different age groups, where immunity dysfunction and cancer related pathways indicated the common theme of aging. Therefore, our results showed the aging process could be predictive by modelling the self-organization system, and indicated crosstalk among aging, immunity and cancers.

Further, the cancer profiles also identified the aging acceleration of cancer samples with aging scores based on the self-organization system being statistically significant. Both methylation and expression profiles found the cancer samples showed aging acceleration compared to normal samples. The results indicated the protective roles of aging in cancers [5]. Moreover, different aging acceleration pattern could also discriminate cancer types.

## Conclusion

In summary, we presented the self-organization model of the aging process based on both methylation and expression profiles in this work, where both ordered and disordered critical patterns were identified in different aging stages. Biological features of the order parameters indicated dysfunction of the immunity system and other common properties during aging (i.e. cancers). Thus the aging acceleration also revealed the relationship between aging and cancers. In conclusion, the aging self-organization system described here is informative to both aging and aging related diseases.

## Materials and methods

### Data and pre-processing

We obtained methylation and expression profiles from MuTHER study [21] and GEO database (https://www.ncbi.nlm.nih.gov/geo/) with the chronological age (Table S5, S6, S7 and S8), respectively. Only profiles in normal tissues of healthy persons were considered for modelling the aging self-organization systems (samples from normal tissues from persons with cancer and disease status sample / blood, e.g. traumatic blood from healthy persons were discarded). As a result, there were 2226 samples of Gene expression data from 37 datasets, and 4428 samples methylation data from 35 datasets were selected to model the aging self-organization systems, respectively.

For each methylation / expression dataset, the data was treated by a Singular Value Decomposition (SVD) method [16, 22] (regress the first 3 principle components) to assess the sources of inter-sample variation separately in each tissue, and then were normalized to have zero mean and unit variance. Finally the profiles were discretized using two thresholds mean+/−std. If the data came from different platform (e.g. GPL96 / GPL97) even in the same GEO Series, or came from different region of brain (e.g. hippocampus, Posterior cingulate region and so on), the data were treated as independent dataset.

The age groups were partitioned as: (0, 20], (2050), [50, 70) and [70, survival). The choice of age groups was guided by the following criterion: first, the partition of methylation and expression data should be accordance for further integration; second, the human methylation “age acceleration” is significant before age of 20 [5]; third, the sample imbalances between age groups need to be small. Data from different age nearby were used to model the aging self-organization system (0–20 vs. 20–50; 20–50 vs. 50–70; 50–70 vs. 70-sruvival) based on methylation and expression profiles, respectively. Further, simplified classification models were also constructed to discriminate “young” (0–50) and “old” (50-survival) age group [6] based on expression and methylation profiles, respectively.

We also downloaded paired methylation, expression, somatic mutation profiles and clinical data (both cancer and adjacent normal tissue) from the TCGA platform (through the xena website: https://xenabrowser.net/hub/) to further analyze aging related genomic alterations (totally 333 paired samples were obtained).

### The computational pipeline of modelling aging self-organization systems

#### Step 1, filtering the aging background network by maximum mutual information minimum redundancy criterion

Aging is a gradual process with biological functional decline / disorder. The degree of disorder is often evaluated by entropy. The aging process is usually accompanied by increasing entropy; however, in the biological non-closed molecular system, particular bio-markers perform special function with ordered patterns in the aging process. Therefore, key order-disorder transitions (or vice versa) accounts towards important changes between age group in the aging process.

In this work, the mutual information between different age groups was used to evaluate relevance between genes (i.e. methylation or expression profiles), and the mutual information between genes (from all training samples / age groups) was used to evaluate gene redundancy. In addition, the background interaction system / network of the aging process satisfied maximum mutual information and minimum redundancy criterion:

1 $$ \mathrm{n} etwork\leftarrow find\left(\sum |{\mathrm{I}}_{group1}^{\left(i,j\right)}-{I}_{group2}^{\left(i,j\right)}|>\sum redundanc{y}^{\left(i,j\right)}\right) $$

where *I*^*(i,j)*^_*group*_ indicates the mutual information between genes within the same age group, evaluating the changed dispersion of gene interaction between age groups by the absolute difference, and *redundancy*^*(i,j)*^ indicates the redundancy between genes. As a result, the mutual information between age groups was filtered by the redundancy in the background network as the preliminary gene interaction system of the aging process.

#### Step 2, summarizing gene interactions by the convolution technology

Each “edge” in the aging background network indicating the changed dispersion of gene interaction. As a result, the entire interaction of a gene could be calculated by convoluting all the edges in the background network (i.e. Fig. 1b), where the absolute differences of mutual information between age groups were used as the convolution kernel.

2 $$ {\mathrm{i}\mathrm{nteraction}}_{\mathrm{i}}=\sum \operatorname{int} eractio{n}_{\left(i,j\right)}\ast \mid \mathit{\operatorname{sign}}\left( gen{e}_i\right)-\mathit{\operatorname{sign}}\left( gen{e}_j\right)\mid $$

where *gene*_*i*_ and *gene*_*j*_ was the profile of i-th and j-th gene, respectively; and

3 $$ {\mathrm{interaction}}_{\left(i,j\right)}=I\left(|\mathit{\operatorname{sign}}\left( gen{e}_i\right)-\mathit{\operatorname{sign}}\left( gen{e}_j\right)|, age\_ group\right) $$

where I(x,y) was the mutual information between x and y.

#### Step 3, calculating the entire pattern by accumulating genes within module

Highly interconnected genes in the network are usually involved in the same biological functions. In this work, genes in the same module were accumulated, where the weights were calculated by the relieff algorithm. Either gene original profiles or convoluted interactions were accumulated was determined by their relieff weights (subtracting the minimum value of the relieff weights). As a result, each module could be a feature in classification of different age groups.

#### Step 4, determining module size by clustering method and cross-validation

The size of module (how many genes within the module) was determined using a hierarchical clustering method, where correlation of genes was evaluated by mutual information in the background network between different age groups in the aging process. The clustering degree / times was determined by (5-fold) cross validation.

#### Step 5, identifying order parameters by network sparsification

In this work, only a small ratio of interactions were convoluted in *step 2*, sorted by the mutual information; and only genes with top relieff values were accumulated in the module in *step 2*. sqrt(n) interactions / genes were selected as the order parameters of the aging process using the network sparsification method, where n was the total number interacted with each gene / within the module, respectively.

In this work, the 0–1 SVM classifier (with the linear kernel) was used to discriminate the age groups. The value of Box Constraint was 1, and the hyperplane was optimized by imposing a penalty on the length of the margin for every observation that is on the wrong side of its class boundary.

### Enrichment analysis

Enrichment analyses were carried out to gain significantly biological functions. GO Biological Processes (BP) terms of Gene Ontology (GO) and KEGG pathways were downloaded from Gene Set Enrichment Analysis (GSEA) platform (version 6.1) [23].

The hypergeometric test [24] was performed to estimate the enrichment of these selected genes compared to known GO terms or pathways. Finally, the selected significant enrichment *p*-values were controlled by False Discovery Rate [25]. The thresholds were set as p-value< 0.05 and FDR < 0.2.

To evaluate annotated functions across, enriched BP terms / KEGG pathways were calculated by summarizing values of 1-fdr, where fdr < 0.2 was set as the threshold.

4 $$ \mathrm{score}=\sum \limits_{fdr<0.2}\left(1- fdr\right) $$

## Supplementary information

**Additional file 1 **: **Figure S1** Hierarchies of the the aging self-organization system. (a, b) cross-talks between the 492th module and other modules in the model of 0–20 vs. 20–50, the methylation profile; (c, d) cross-talks between the 1799th module and other modules in the model of 20–50 vs. 50–70, the expression profile; (a, c) enriched BP terms; (b, d) enriched KEGG pathways; **Figure S2** Age acceleration versus number of somatic mutations in the TCGA data based on methylation profiles. **Figure S3** Age acceleration versus number of somatic mutations in the TCGA data based on expression profiles. **Figure S4** aging acceleration characteristics across cancers using the top differential expression module. (a) connection of BP terms based on order-parameter modules; (b) connection of KEGG pathways based on order-parameter modules;

**Additional file 2 **: **Table S1** modules based on order-parameters of the aging self-organization system using methylation profiles. (XLS 2406 kb)

**Additional file 3 **: **Table S2** modules based on order-parameters of the aging self-organization system using expression profiles. (XLS 1322 kb)

**Additional file 4 **: **Table S3** other classification results in methylation profiles. **Table S4** other classification results in expression profiles. **Table S5** gene expression data involving normal tissues from healthy persons. **Table S6** DNA methylation data involving normal tissues from healthy persons.

**Additional file 5 **: **Table S7** data partition of gene expression profiles as well as the age (XLS 130 kb)

**Additional file 6 **: **Table S8** data partition of DNA methylation profiles as well as the age. **Note**: 0 indicates genes are not selected as order-parameters, otherwise are selected within modules (Table S1-S2). 0 stands for training data, and 1 stands for test data in the third column (Table S7-S8). (XLS 242 kb)

## Data Availability

The data supporting the results of this article are included and cited within the article and its additional files.
