# Supplementary material for: The self-organization model reveals systematic characteristics of aging
Source: Theor Biol Med Model. 2020 Mar 20;17:4. doi: 10.1186/s12976-020-00120-z (PMC7082995; doi:10.1186/s12976-020-00120-z)
Supplement: Supplementary file 1 — Additional file 1 : Figure S1 Hierarchies of the the aging self-organization system. (a, b) cross-talks between the 492th module and other modules in the model of 0–20 vs. 20–50, the methylation profile; (c, d) cross-talks between the 1799th module and other modules in the model of 20–50 vs. 50–70, the expression profile; (a, c) enriched BP terms; (b, d) enriched KEGG pathways; Figure S2 Age acceleration versus number of somatic mutations in the TCGA data based on methylation profiles. Figure S3 Age acceleration versus number of somatic mutations in the TCGA data based on expression profiles. Figure S4 aging acceleration characteristics across cancers using the top differential expression module. (a) connection of BP terms based on order-parameter modules; (b) connection of KEGG pathways based on order-parameter modules; [file 12976_2020_120_MOESM1_ESM.doc]

**Supplemental Files**


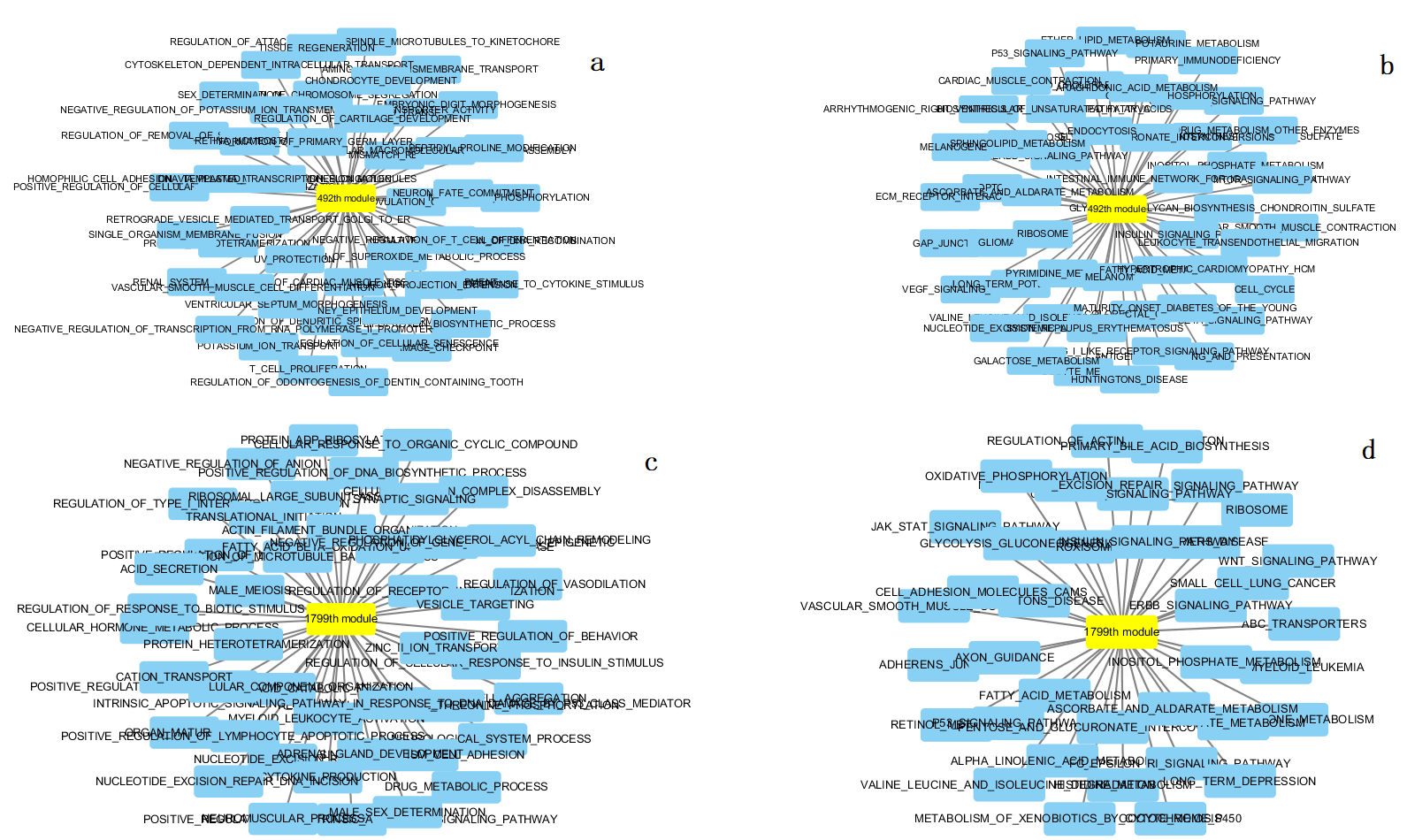


**Figure S1** Hierarchies of the the aging self-organization system.

(a, b) cross-talks between the 492th module and other modules in the model of 0-20 vs. 20-50; (c, d) cross-talks between the 1799th module and other modules in the model of 20-50 vs. 50-70; (a, c) enriched BP terms; (b, d) enriched KEGG pathways;


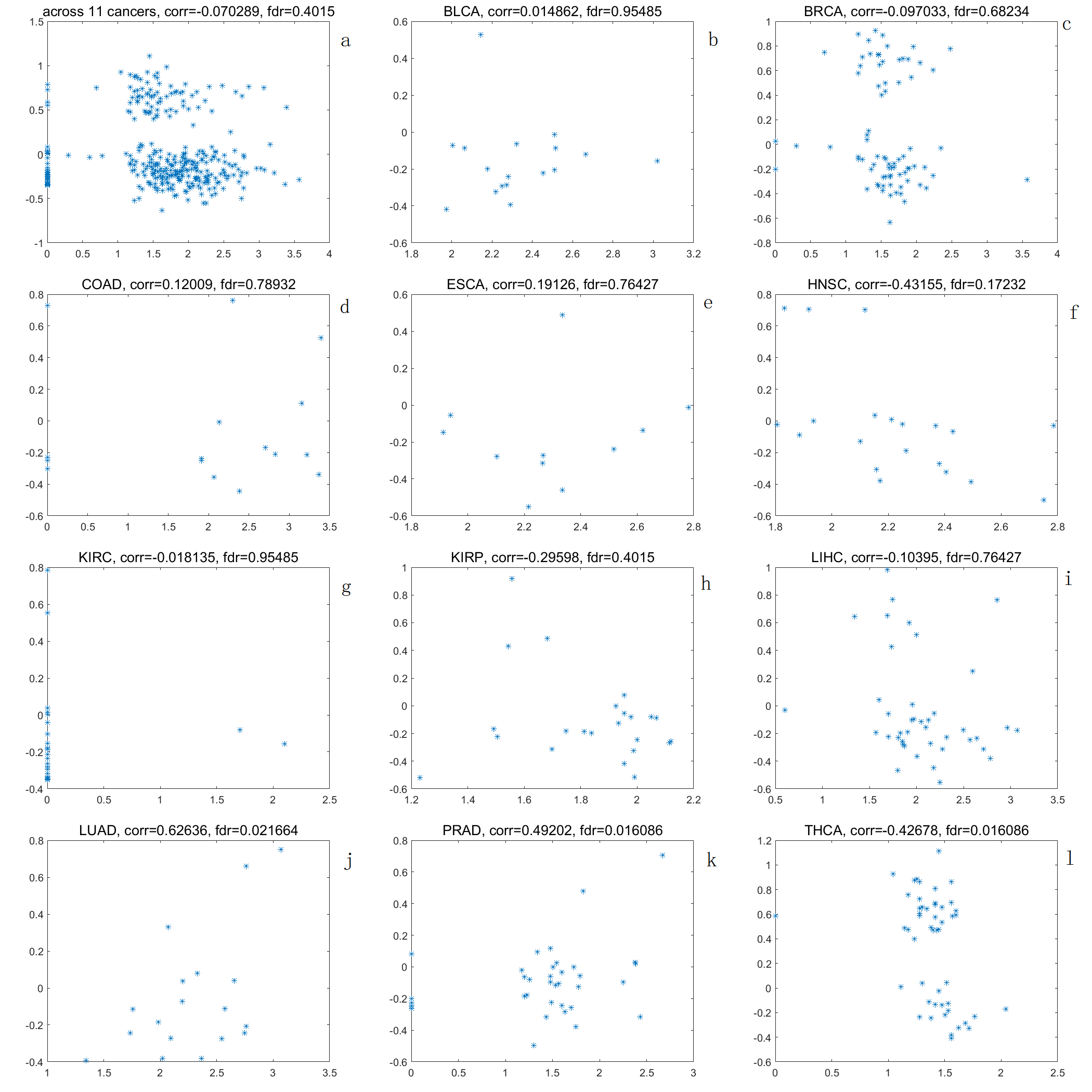


**Figure S2** Age acceleration versus number of somatic mutations in the TCGA data based on methylation profiles


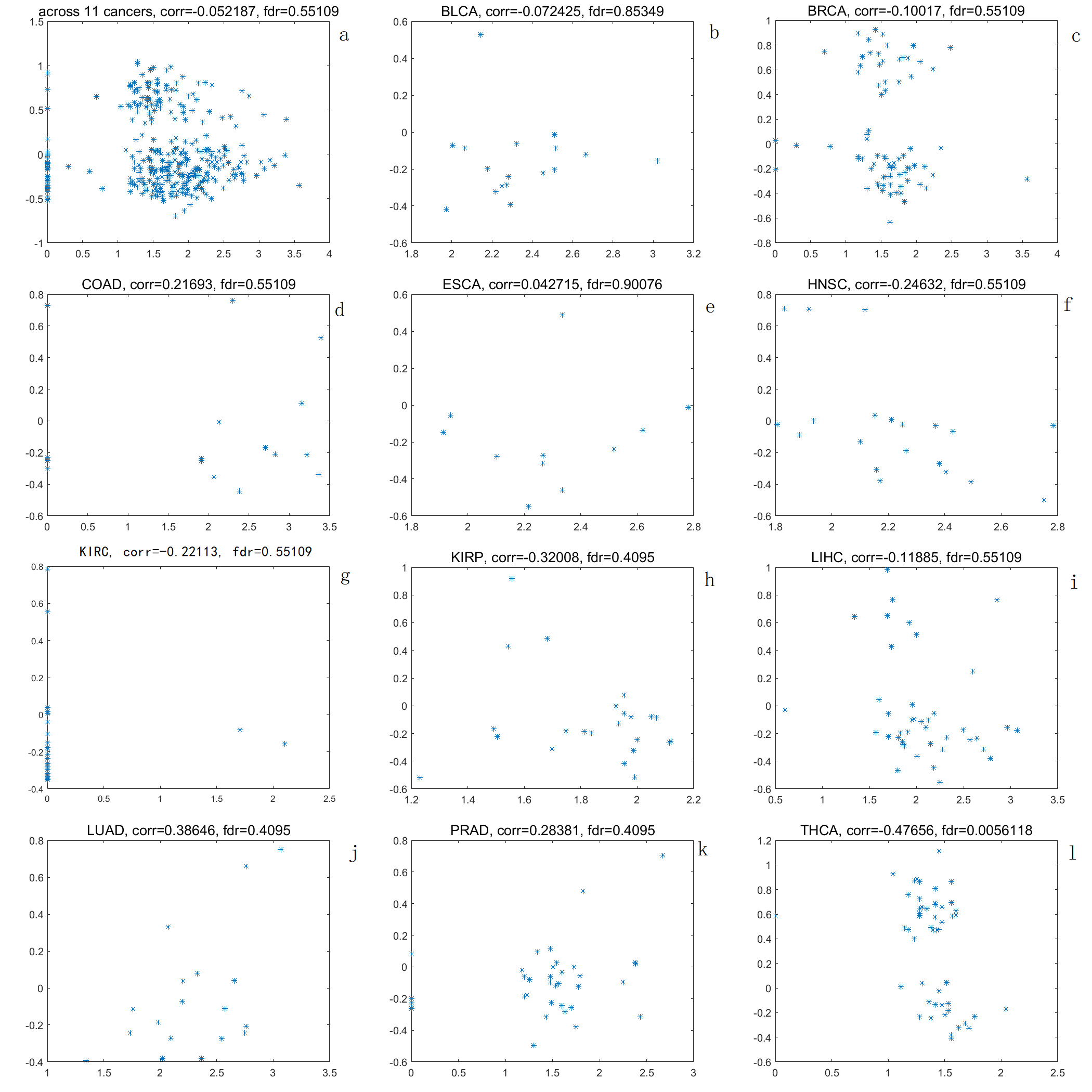


**Figure S3** Age acceleration versus number of somatic mutations in the TCGA data based on methylation profiles


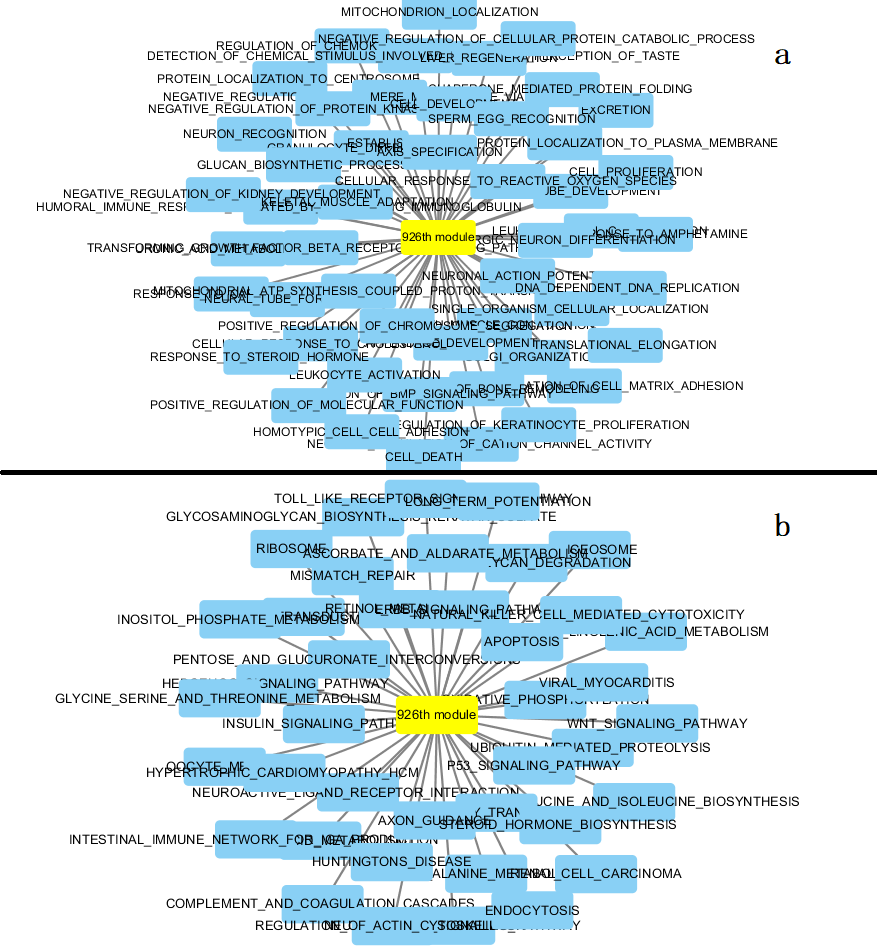


**Figure S4** aging acceleration characteristics across cancers using the top differential expression module.

(a) connection of BP terms based on order-parameter modules; (b) connection of KEGG pathways based on order-parameter modules;
