# Supplementary material for: The self-organization model reveals systematic characteristics of aging
Source: Theor Biol Med Model. 2020 Mar 20;17:4. doi: 10.1186/s12976-020-00120-z (PMC7082995; doi:10.1186/s12976-020-00120-z)
Supplement: Supplementary file 4 — Additional file 4 : Table S3 other classification results in methylation profiles. Table S4 other classification results in expression profiles. Table S5 gene expression data involving normal tissues from healthy persons. Table S6 DNA methylation data involving normal tissues from healthy persons. [file 12976_2020_120_MOESM4_ESM.doc]

**Table S3** classification results (error rates) based on methylation data

| Age group | Training data(NNA) | Test data (NNA) | Training data(NB) | Test data (NB) |
| --- | --- | --- | --- | --- |
| 0-20 vs. 20-50 | 0.4662 | 0.4847 | 0.4784 | 0.4885 |
| 20-50 vs. 50-70 | 0.4656 | 0.476 | 0.3938 | 0.3741 |
| 50-70 vs. 70-survival | 0.4527 | 0.457 | 0.4365 | 0.4615 |
| 0-50 vs. 50-survival | 0.4594 | 0.4613 | 0.3934 | 0.3724 |

**Table S4** classification results (error rates) based on expression data

| Age group | Training data(NNA) | Test data (NNA) | Training data(NB) | Test data (NB) |
| --- | --- | --- | --- | --- |
| 0-20 vs. 20-50 | 0.3044 | 0.3838 | 0.3044 | 0.2645 |
| 20-50 vs. 50-70 | 0.2915 | 0.2917 | 0.2238 | 0.2021 |
| 50-70 vs. 70-survival | 0.3268 | 0.3437 | 0.2333 | 0.2496 |
| 0-50 vs. 50-survival | 0.3438 | 0.3391 | 0.2334 | 0.2036 |

Table S5 Gene expression data involving normal tissues from healthy persons

| No. | Data | tissue | samples |
| --- | --- | --- | --- |
| 1 | MuTHER study | fat | 193 |
| 2 | MuTHER study | skin | 188 |
| 3 | MuTHER study | LCL | 185 |
| 4 | GSE362 | Muscle | 15 |
| 5 | GSE362 | Muscle | 15 |
| 6 | GSE5281 | Entorhinal Cortex | 13 |
| 7 | GSE5281 | hippocampus | 13 |
| 8 | GSE5281 | Medial Temporal Gyrus | 12 |
| 9 | GSE5281 | Posterior Cingulate | 13 |
| 10 | GSE5281 | Superior Frontal Gyrus | 11 |
| 11 | GSE5281 | Primary Visual Cortex | 12 |
| 12 | GSE22688 | endothelial cell | 38 |
| 13 | GSE29801 | macular Retina | 50 |
| 14 | GSE29801 | extramacular RPE-choroid | 46 |
| 15 | GSE29801 | macular RPE-choroid | 28 |
| 16 | GSE29801 | extramacular Retina | 27 |
| 17 | GSE37171 | Whole blood | 40 |
| 18 | GSE43973 | breast | 71 |
| 19 | GSE38959 | normal mammary gland ductal cells | 13 |
| 20 | GSE1572 | frontal cortex | 30 |
| 21 | GSE5388 | dorsolateral prefrontal cortex | 31 |
| 22 | GSE5389 | orbitofrontal cortex | 11 |
| 23 | GSE8919 | frontal cortex | 42 |
| 24 | GSE8919 | temporal cortex | 143 |
| 25 | GSE3790 | cerebellum | 32 |
| 26 | GSE3790 | frontal cortex | 28 |
| 27 | GSE3790 | caudate nucleus | 27 |
| 28 | GSE3790 | cerebellum | 32 |
| 29 | GSE3790 | frontal cortex | 29 |
| 30 | GSE3790 | caudate nucleus | 28 |
| 31 | GSE58015 | Dendritic cells | 9 |
| 32 | GSE77164 | Whole blood | 225 |
| 33 | GSE52699 | myoblasts | 14 |
| 34 | GSE15745 | cerebellum | 124 |
| 35 | GSE15745 | frontal cortex | 146 |
| 36 | GSE15745 | pons | 145 |
| 37 | GSE15745 | temporal cortex | 147 |

Table S6 DNA methylation data involving normal tissues from healthy persons

| Data | tissue | samples |
| --- | --- | --- |
| GSE15745 | cerebellum | 355 |
| GSE15745 | frontal cortex | 369 |
| GSE15745 | pons | 125 |
| GSE15745 | cerebellum | 127 |
| GSE17448 | Mesenchymal Stromal Cells | 16 |
| GSE19711 | Peripheral Whole Blood | 274 |
| GSE20067 | Whole blood | 100 |
| GSE20236 | blood | 93 |
| GSE20242 | CD4+ T-cells | 50 |
| GSE22595 | Dermal Fibroblasts | 15 |
| GSE25892 | epithelial cell | 109 |
| GSE27317 | Umbilical cord blood | 168 |
| GSE30758 | uterine cervix | 102 |
| GSE30870 | CD4+ cell | 20 |
| GSE30870 | PBMNC | 20 |
| GSE32146 | Colon mucosa | 15 |
| GSE32148 | peripheral blood | 31 |
| GSE32393 | breast | 114 |
| GSE34035 | saliva | 197 |
| GSE34257 | Umbilical cord blood | 84 |
| GSE34639 | CD4+ cell | 48 |
| GSE36642 | cord blood mononuclear cells | 95 |
| GSE36642 | umbilical vascular endothelial cells | 28 |
| GSE36812 | cord blood | 48 |
| GSE37008 | PBMC | 99 |
| GSE38608 | cerebellar | 17 |
| GSE38873 | cerebellum | 118 |
| GSE40279 | Whole blood | 656 |
| GSE41037 | Whole blood | 395 |
| GSE41169 | Whole blood | 33 |
| GSE41826 | post mortem frontal cortex | 77 |
| GSE42700 | Buccal cell | 53 |
| GSE42861 | peripheral blood leukocytes | 336 |
| GSE43269 | knee cartilage | 18 |
| GSE44667 | placental chorionic villus | 40 |
